# [Retracted] Norcantharidin induces ferroptosis via the suppression of NRF2/HO‑1 signaling in ovarian cancer cells

**DOI:** 10.3892/ol.2025.14861

**Published:** 2025-01-07

**Authors:** Xiaoyan Zhu, Xiaohong Chen, Longshan Qiu, Jianhua Zhu, Jiancai Wang

Oncol Lett 24: W359, 2022; DOI: 10.3892/ol.2022.13479

Following the publication of this paper, it was drawn to the Editor's attention by a concerned reader that certain of the DCFH-DA-stained cellular data shown in Fig. 2C and D, the flow cytometric data in Fig. 2E and F, and western blotting data shown in Figs. 4C and 5H were strikingly similar to data that had either already appeared in previously published articles written by different authors at different research institutes (one of which has since been retracted), or were featured in an article that was submitted for publication to a different journal at around the same time. Owing to the fact that the contentious data in the above article had already been published prior to its submission to *Oncology Letters*, the Editor has decided that this paper should be retracted from the Journal. After having been in contact with the authors, they accepted the decision to retract the paper. The Editor apologizes to the readership for any inconvenience caused.

